# It's about time: studying gene regulatory programs across serial organs

**DOI:** 10.1186/s13059-017-1166-6

**Published:** 2017-02-16

**Authors:** Maayan Baron, Itai Yanai

**Affiliations:** Institute for Computational Medicine, New York University School of Medicine, New York, USA

Underlying the process of development is an intricate gene regulatory program that involves modules of genes invoked at different times and in different spaces throughout an organism. As an obvious set of examples, the patterning of our eyes, ears, limbs, and teeth results from such iteratively deployed gene regulatory programs [1]. The similarities among these serial organs allow us to presume that the programs invoked are essentially identical. In some serial organs, however, important differences are also manifested, as in the cases of our arms and legs, and our different types of teeth. Thus, an important question is how variation across these organs is encoded in gene regulatory programs.

**Fig. 1:**
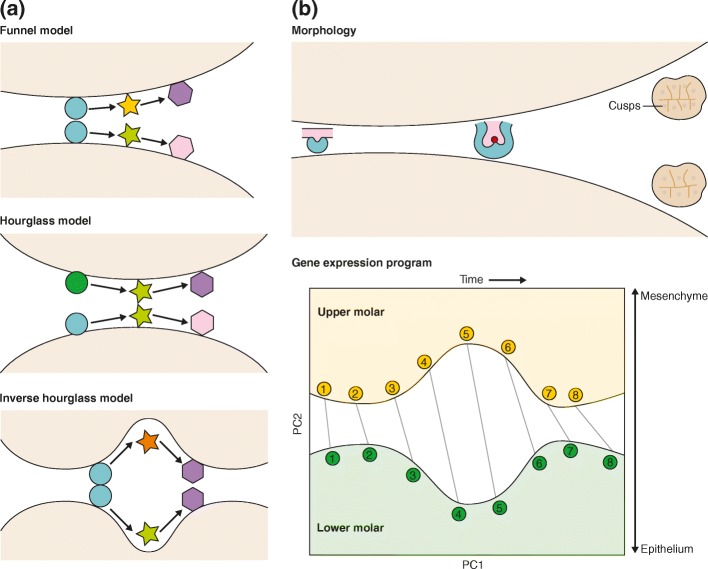
Gene regulatory programs across serial organs. **a** Possible explanations for distinct serial organ programs. The beginning, the middle, and the ends of development are most conserved in the funnel, hourglass, and inverse hourglass models, respectively. **b** Morphologically, the mouse upper and lower molars are similar at the beginning of development but diverge as development progresses (*upper panel*). Their gene expression programs, however, are most distinct in the middle of their development. The largest axis of variation (the first principal component (*PC1*)) corresponds to time, and the second largest (the second principal component (*PC2*)) is consistent with the proportions of constituent tissue types (*lower panel*)

The gene regulatory programs of serial organs are clearly homologous in the sense that they are derived from modified versions of an ancestral program. In a recent study by Pantalacci et al. [2], published by Genome Biology, the authors investigated whether the differences among a pair of serial organ gene profiles—those of an upper and lower jaw tooth—are concentrated at the ends of the program, while an intermediate part is conserved. Such an hourglass model (Fig. 1a) would suggest that increased developmental constraints occur in the middle of the developmental process or that the ends of the program are most adaptive. Alternatively, differences in gene regulation may be concentrated at the end of the process, leading to a funnel-like model (Fig. 1a) that reflects the pattern of morphological differences among these teeth, which become increasingly different as development progresses. Finally, the requisite rewiring could be concentrated in the middle of development, leading to a model with an “inverse-hourglass-like” shape (Fig. 1a).

Interestingly, such a question is reminiscent of the comparisons made among species within and across phyla [3, 4]. By comparing the gene regulatory programs of serial organs, one benefits from the exclusion of the effect of different genotypes (across species) and can ask how different organs result from the expression of the same set of genes. Barbara McClintock has been quoted saying “if I could control the time of gene action, I could cause a fertilized snail egg to develop into an elephant” [5]. The differences among serial organs make this point even more compelling: from the same set of genes we get different types of teeth.

Teeth are serial organs that develop from a combination of epithelial and mesenchymal tissues. Mouse tooth development is well-studied and begins when an epithelial tissue induces mesenchyme growth, leading to bud formation. The developmental processes for distinct types of teeth—such as incisors, canines, premolars, and molars—have unique aspects that derive from their unique local pathways, but all teeth share some global developmental patterns. The epithelial–mesenchymal interactions that occur during tooth development are regulated by genes that are conserved across vertebrates [6]. However, although teeth are common to most vertebrates, molars—which develop from simpler teeth—are unique to mammals. Molar development begins with epithelial thickening, which results from cell proliferation. The proliferating epithelium and the mesenchymal tissue form a bud, and subsequently a primary enamel knot is formed during the cap stage. The complex structure of the molar tooth is created when the epithelium folds around the mesenchyme, forming a bell, which is of a different structure for different types of molar across the upper and lower jaws. While such morphological observations are revealing, it is of acute interest to understand the molecular mechanisms that distinguish the different types of molar.

The transcriptome constitutes the first phenotype of the genome. RNA sequencing (RNA-seq) allows for systematic unbiased inquiry of the transcriptome and reveals expression levels across cells, tissues, and embryos, as well as both within and across species [4, 7]. Although transcriptome-level expression does not perfectly reflect protein levels, it is highly dynamic and provides a resolved picture of the transcript upregulations and downregulations that characterize tissues and cells. To compare the development of the upper and lower first molars in mice, Pantalacci et al. studied eight developmental time-points using RNA-seq and investigated the extent of similarities and differences between these two serial organs. The authors found that the largest component of variation among the samples was time (Fig. 1b, lower panel), as generally seen in developmental transcriptomes [8]. The second largest component of variation across the time-points, however, corresponded to the physical location of the molar tooth: that is, whether it was in the upper or lower jaw (Fig. 1b, lower panel). This is intriguing since, overall, a strong similarity between the two developmental processes was observed. Surprisingly, even when “lower jaw” and “upper jaw” marker genes were excluded, the difference was still detectable, leading the authors to discover that the differences were due to the proportions of mesenchymal and epithelial tissue that comprised each type of tooth. While both upper and lower molars contain mesenchymal and epithelial tissues, the upper molar developmental program more closely resembles a pure mesenchymal program, whereas the lower molar developmental program more closely resembles a pure epithelial program. The authors’ analysis indicated that variation is largest in the middle stages of the development of the two types of molar—which corresponds to the inverse hourglass model (Fig. 1a)—during which the differences in expression correspond to genes involved in cell migration and cell adhesion during crown morphogenesis.

The upper molar and lower molar gene regulatory programs also differed according to the timing of their expression. The authors observed that the upper molar program was consistently in a less advanced state than that of the lower molar. Such heterochrony—a term that refers to a change in the timing of expression of a phenotypic trait—is reminiscent of what is observed across species [9]. Heterochrony has been shown to produce morphological novelties, which suggests that various developmental processes may be reprogrammed by shifts in the relative timings of gene expression [10]. Indeed, the heterochrony between upper and lower molars is interesting because it may be the source of differences in the number of cusps that they contain (Fig. 1b, upper panel). As upper molars are in a less-differentiated state for a longer period of time than are lower molars, upper molars may develop additional cusps. This phenomenon may even extend further; when implementing this approach to analyze a previously reported dataset from a study of forelimb and hindlimb development, Pantalacci et al. again found that the main difference was due to the proportion of cartilage tissue comprising the limb. Moreover, they detected heterochrony in the expression programs of forelimb and hindlimb development: the forelimb was relatively more mature than was the hindlimb. Collectively, the work by Pantalacci et al. sheds light on differences in the development of serial organs and reveals that those differences are mainly due to heterochrony and the proportions of different tissue types that comprise the organ.

As interactions among tissues are pivotal for tooth development, it will be interesting to explore the extent of the differences in mesenchymal tissue across different types of teeth using single-cell RNA-seq. Applying this technology will help us to better characterize differences between tissue gene expression programs by identifying any subpopulations within each tissue and will also enable further characterization of differences in the proportions of constituent tissue types. It may also be interesting to study the interactions that occur between specific cells from different tissues. For example, examining the interactions that occur between mesenchymal and epithelial cells may help us to understand the signals that regulate tooth development and reveal the reprogramming that is required to distinguish serial organs.
